# The silent epidemic: domestic violence and its devastating impact on women's health

**DOI:** 10.1590/1806-9282.2025718EDIT

**Published:** 2025-09-19

**Authors:** José Maria Soares, Débora Krakauer, Edmund Chada Baracat

**Affiliations:** 1 Disciplina de Ginecologia, Departamento de Obstetrícia e Ginecologia, Hospital das Clínicas, Faculdade de Medicina da Universidade de São Paulo (FMUSP) – São Paulo (SP), Brazil.

Domestic violence against women remains one of the most pervasive yet underaddressed public health crises of our time^1^. Recent research from the University of São Paulo sheds new light on the profound health consequences faced by survivors, particularly women in the climacteric phase of life^2^. The study's findings demand urgent attention from healthcare providers, policymakers, and society at large, revealing how violence transcends social boundaries to become embedded in women's biological and psychological well-being^3^.

The research compared 100 women aged 40–65 years, with half being victims of domestic violence^2^. The results were staggering: women who experienced abuse showed significantly higher rates of chronic pelvic pain and sexual dysfunction compared to the control group^2^. These physical manifestations were accompanied by alarming rates of post-traumatic stress disorder, depression, and anxiety^4,5^. The data paints a clear picture of how violence doesn't end when the physical abuse stops, but rather becomes metabolized into women's very physiology^6^.

What makes these findings particularly troubling is the window they provide into the long-term health consequences of domestic violence^7^. The studied population—women in midlife—demonstrates how early experiences of abuse can manifest decades later as chronic health conditions^8^. The high prevalence of hypertension and diabetes among survivors suggests that the toxic stress of violence may accelerate biological aging and increase vulnerability to metabolic disorders^9^. This challenges our conventional timelines for intervention, emphasizing the need for lifelong support systems^10^.

The sexual health findings reveal another layer of this complex issue^4^. While both groups reported sexual difficulties common to menopausal women, the abuse survivors showed markedly higher rates of vaginismus and dyspareunia^2^. This suggests that the trauma of violence becomes somatized in women's sexual function, creating barriers to intimacy that persist long after leaving abusive relationships^6^. The psychological scars of coercion and violation appear to rewrite neural pathways related to pleasure and pain^5^.

Perhaps most alarming is the study's revelation about healthcare engagement^2^. Despite their significant health burdens, only 28% of abuse survivors were receiving psychological support^4^. This gap highlights systemic failures in our healthcare infrastructure to identify and properly care for victims of domestic violence^7^. The medical community's traditional separation of physical and mental health services creates dangerous blind spots when treating trauma survivors^8^.

The implications for clinical practice are profound^3^. Healthcare providers must adopt universal screening protocols for domestic violence across all specialties, particularly in gynecology and primary care settings^9^. Medical education needs to better equip practitioners to recognize the subtle presentations of trauma in patients’ health histories^10^. We must move beyond simply treating symptoms to addressing their root causes in violence and abuse^1^.

On a policy level, these findings underscore the need for integrated care models that bridge medical, psychological, and social services^7^. The traditional silos of healthcare delivery fail abuse survivors, who require coordinated support across multiple domains^8^. Public health initiatives should prioritize trauma-informed care training and develop clear referral pathways between medical facilities and social support services^9^.

The economic argument for intervention is equally compelling^10^. The chronic health conditions developed by abuse survivors represent a significant burden on healthcare systems^2^. Preventive measures and early interventions could potentially save millions in long-term treatment costs while dramatically improving quality of life^4^. This study provides the evidence base for such investments in women's health infrastructure^6^.

As a society, we must confront the uncomfortable truth that domestic violence is not merely a private matter or criminal issue, but a determinant of population health^3^. The biomedical markers of abuse survivors—from elevated inflammatory markers to altered stress responses—demonstrate how social violence becomes a biological reality^5^. This demands a public health response proportionate to the scale of the problem^7^.

The time for passive acknowledgment has passed^1^. This research provides irrefutable evidence that domestic violence is a medical emergency with lifelong consequences^2^. As healthcare professionals, we have both the opportunity and obligation to transform these findings into action—through better screening, more compassionate care, and stronger advocacy for policies that protect women's health^9^. The women in this study have given us their pain as data; we owe them more than just publication of results—we owe them change^10^.
